# Supplementary material for: Effects of Exogenous Melatonin on Root Physiology, Transcriptome and Metabolome of Cotton Seedlings under Salt Stress
Source: Int J Mol Sci. 2022 Aug 21;23(16):9456. doi: 10.3390/ijms23169456 (PMC9409268; doi:10.3390/ijms23169456)
Supplement: Supplementary file 1 [file ijms-23-09456-s001.zip › Figures.pdf]

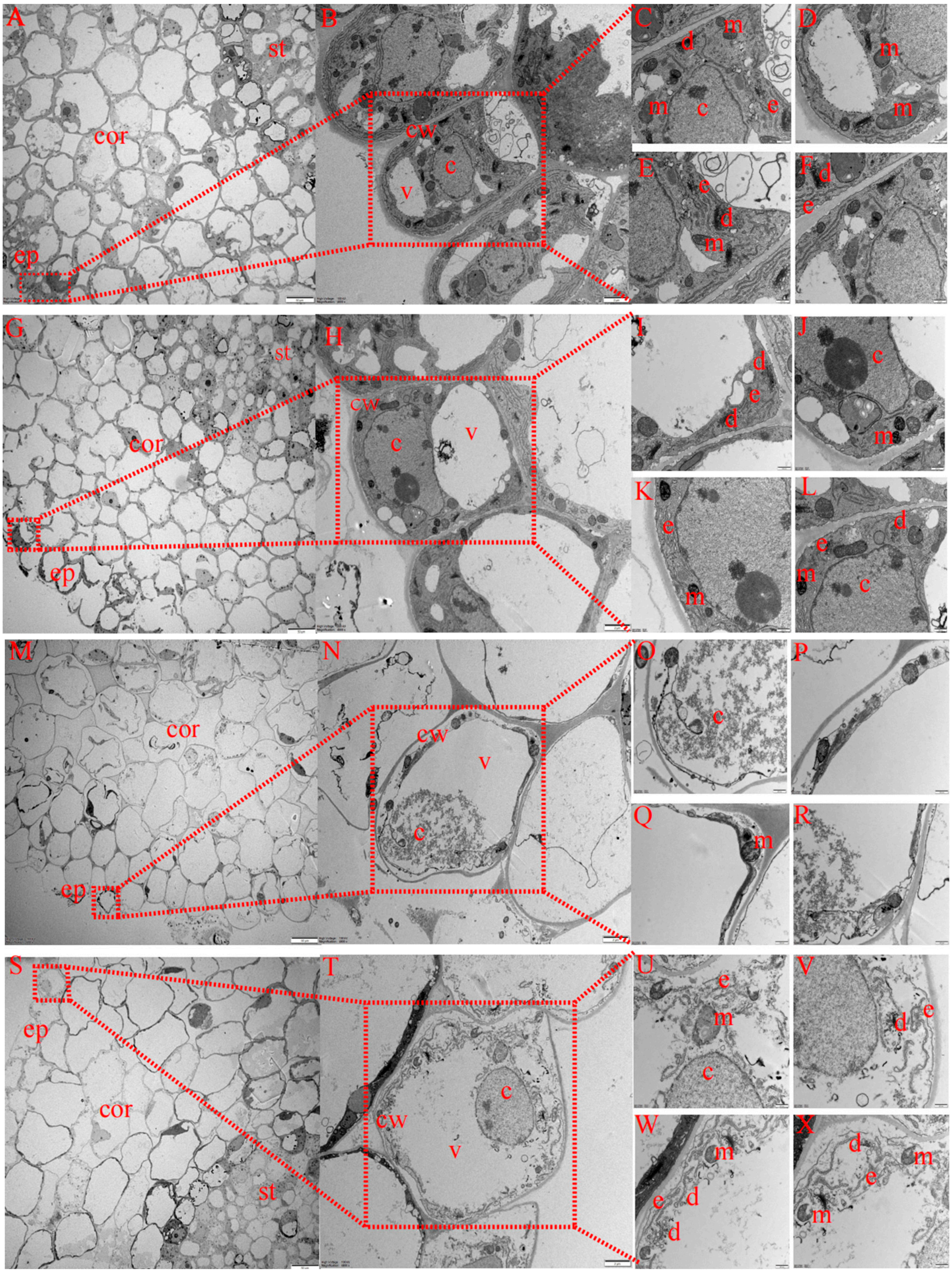

Fig. S1 Effects of melatonin on the ultrastructure of root epidermis cells of cotton seedlings under salt stress. (A – F) figure shows the TEM structure of epidermisl cell of CK; (G – L) figure shows the TEM structure of epidermisl cell of MT; (M-R) figure shows figure shows the TEM structure of epidermisl cell of S; (S-X) figure shows figure shows the TEM structure of epidermisl cell of MS; ep:epidermis; cor: cortex; st: stele; m: mitochondria; e: endoplasmic reticulum; d:dictyosome; c: cell nucleus; v: vacuole.

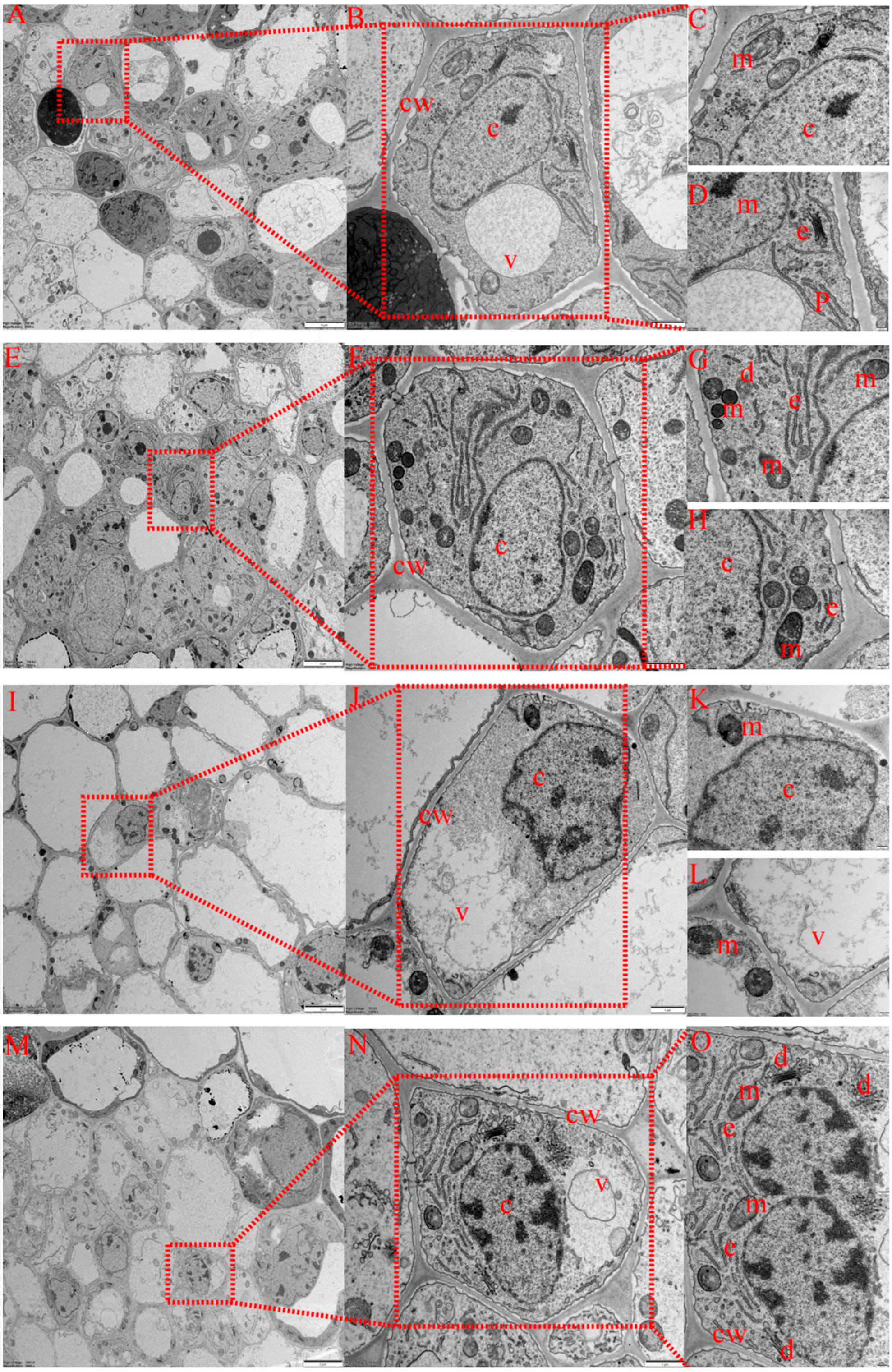

Fig. S2 Effects of melatonin on the ultrastructure of root stele cells of cotton seedlings under salt stress.

(A－D) figure shows the TEM structure of stele cell of CK; (E－H) figure shows the TEM structure of stele cell of MT; (I-L) figure shows figure shows the TEM structure of stele cell of S; (M-P) figure shows figure shows the TEM structure of stele cell of MS; ep:epidermis; cor: cortex; st: stele; m: mitochondria; e: endoplasmic reticulum; d:dictyosome; c: cell nucleus; v: vacuole.

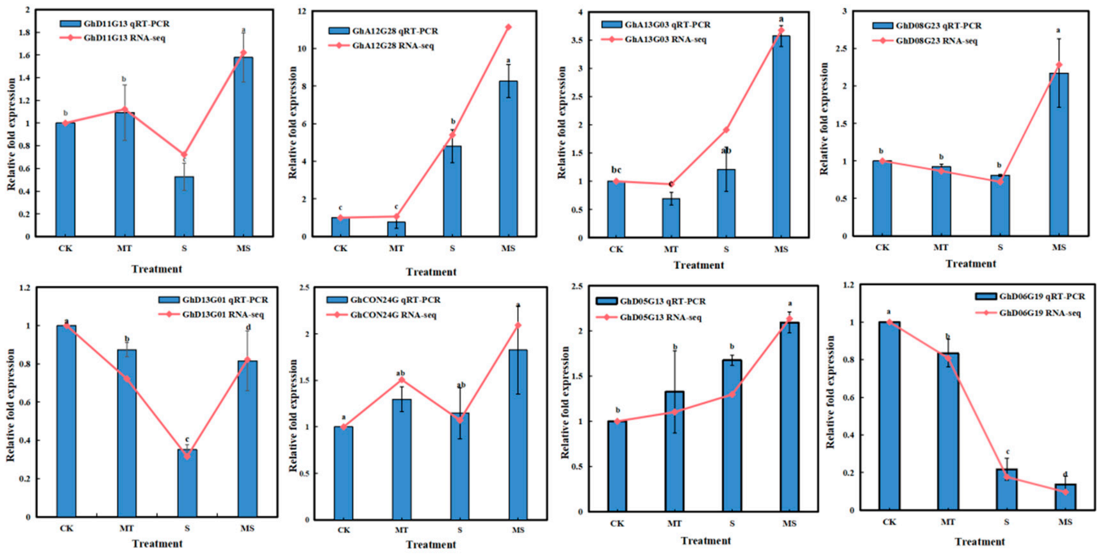

Figure S3. qRT-PCR expression data for roots of cotton seedlings under control (CK), melatonin (MT), NaCl (S) and combined melatonin and NaCl (MS) treatments.

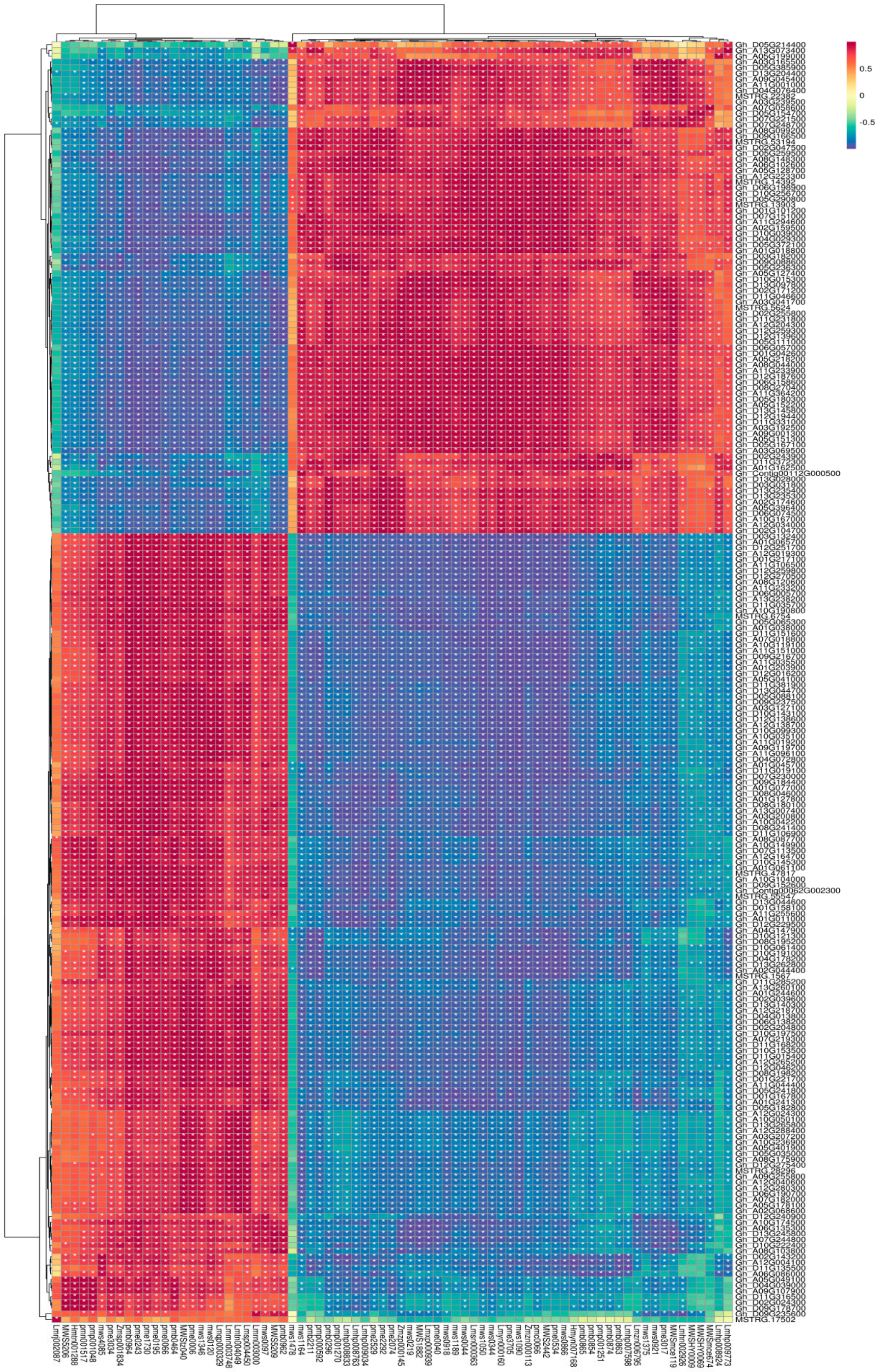

Fig. S4 - Correlation analysis of differentially expressed genes and differentially accumulated metabolites. The absolute value of correlation coefficient is greater than 0.5 and the top 250 differential genes and differential metabolites are used for correlation analysis.
