# Supplementary material for: Effects of Exogenous Melatonin on Root Physiology, Transcriptome and Metabolome of Cotton Seedlings under Salt Stress
Source: Int J Mol Sci. 2022 Aug 21;23(16):9456. doi: 10.3390/ijms23169456 (PMC9409268; doi:10.3390/ijms23169456)
Supplement: Supplementary file 1 [file ijms-23-09456-s001.zip › Tables.pdf]

Table S1 Primer design sequence of eight Quantitative Genes and biological function of genes subjected to qRT-PCR

**Gene ID**	**Sequence (5'-3')**	Pathway annotation
Gh_A12G288400	Forward primer CTGGAAGCCGGTGATCTGTT<br>Reverse primer GGCCCGTTATTCAATTCCGC	MAPK signaling pathway - plant; protein phosphatase 2C
Gh_A13G037600	Forward primer ACCTCGATCTTGCATTGGCA<br>Reverse primer AGTGTAAGACGTGGTGCAGG	Brassinosteroid biosynthesis
Gh_Contig00024G000100	Forward primer TGTACTGGCGGAAATGCTGT<br>Reverse primer GAGGCATGCAATGAGCTTCG	Metabolic pathways; monodehydroascorbate reductase
Gh_D05G131400	Forward primer ATACAGCTGTGCCTCCCAAC<br>Reverse primer GGGAGCACACTTGGTGATGA	Plant hormone signal transduction; auxin-responsive protein IAA
Gh_D06G191100	Forward primer CAACAACCTCCACCCCCTAC<br>Reverse primer GGTTAGGACCCAAGGTGTGG	MAPK signaling pathway - plant; abscisic acid receptor PYR/PYL family
Gh_D08G239500	Forward primer CCTTTGCTGCTTTGCTTCGT<br>Reverse primer CGCCAGCAGCTCAAACAAAT	Metabolic pathways; COMT
Gh_D11G139300	Forward primer TTGCTGGGACGACCTTCTTC<br>Reverse primer GGTGGAGTTGGACTCTGTGG	Plant hormone signal transduction; ethylene-responsive transcription factor 1
Gh_D13G014400	Forward primer CATCCTGCGTGAAGGGCTTA<br>Reverse primer CGAAAGGCCTGTCCACTGAT	Zeatin biosynthesis; cytokinin dehydrogenase

Table S2 KEGG pathway enrichment results.

	KEGG_A_class	KEGG_B_class	Pathway	Pvalue	Pathway ID
MT-vs-CK	Metabolism	Biosynthesis of other secondary metabolites	Phenylpropanoid biosynthesis	0.0072	ko00940
	Metabolism	Energy metabolism	Photosynthesis	0.0193	ko00195
	Metabolism	Metabolism of cofactors and vitamins	Folate biosynthesis	0.0206	ko00790
	Metabolism	Global and overview maps	Biosynthesis of secondary metabolites	0.0332	ko01110
	Metabolism	Biosynthesis of other secondary metabolites	Stilbenoid, diarylheptanoid and gingerol biosynthesis	0.0377	ko00945
	Metabolism	Lipid metabolism	Ether lipid metabolism	0.0410	ko00565
S-vs-CK	Metabolism	Global and overview maps	Biosynthesis of secondary metabolites	0.0000	ko01110
	Metabolism	Biosynthesis of other secondary metabolites	Phenylpropanoid biosynthesis	0.0000	ko00940
	Metabolism	Global and overview maps	Metabolic pathways	0.0000	ko01100
	Metabolism	Lipid metabolism	Cutin, suberine and wax biosynthesis	0.0000	ko00073
	Metabolism	Metabolism of terpenoids and polyketides	Carotenoid biosynthesis	0.0000	ko00906
	Environmental Information Processing	Signal transduction	MAPK signaling pathway - plant	0.0000	ko04016
	Environmental Information Processing	Signal transduction	Plant hormone signal transduction	0.0000	ko04075
	Metabolism	Carbohydrate metabolism	Starch and sucrose metabolism	0.0002	ko00500
	Metabolism	Metabolism of terpenoids and polyketides	Sesquiterpenoid and triterpenoid biosynthesis	0.0004	ko00909
	Metabolism	Lipid metabolism	Glycerophospholipid metabolism	0.0005	ko00564
	Metabolism	Carbohydrate metabolism	Galactose metabolism	0.0000	ko00052

MS-vs-S	Metabolism	Global and overview maps	Biosynthesis of secondary metabolites	0.0000	ko01110
	Metabolism	Global and overview maps	Metabolic pathways	0.0000	ko01100
	Environmental Information Processing	Signal transduction	MAPK signaling pathway - plant	0.0000	ko04016
	Metabolism	Biosynthesis of other secondary metabolites	Phenylpropanoid biosynthesis	0.0005	ko00940
	Metabolism	Carbohydrate metabolism	Pentose phosphate pathway	0.0152	ko00030
	Metabolism	Biosynthesis of other secondary metabolites	Glucosinolate biosynthesis	0.0222	ko00966
	Metabolism	Metabolism of terpenoids and polyketides	Sesquiterpenoid and triterpenoid biosynthesis	0.0248	ko00909
	Metabolism	Carbohydrate metabolism	Glycolysis / Gluconeogenesis	0.0252	ko00010
	Environmental Information Processing	Signal transduction	Plant hormone signal transduction	0.0270	ko04075
MS-vs-MT	Metabolism	Global and overview maps	Biosynthesis of secondary metabolites	0.0000	ko01110
	Metabolism	Biosynthesis of other secondary metabolites	Phenylpropanoid biosynthesis	0.0000	ko00940
	Metabolism	Lipid metabolism	Cutin, suberine and wax biosynthesis	0.0000	ko00073
	Metabolism	Global and overview maps	Metabolic pathways	0.0000	ko01100
	Environmental Information Processing	Signal transduction	Plant hormone signal transduction	0.0000	ko04075
	Environmental Information Processing	Signal transduction	MAPK signaling pathway - plant	0.0000	ko04016
	Metabolism	Metabolism of terpenoids and polyketides	Carotenoid biosynthesis	0.0000	ko00906
	Metabolism	Lipid metabolism	Glycerolipid metabolism	0.0000	ko00561
	Metabolism	Metabolism of terpenoids and polyketides	Zeatin biosynthesis	0.0001	ko00908
	Metabolism	Biosynthesis of other	Stilbenoid,	0.0005	ko00945

	secondary metabolites	diarylheptanoid and gingerol biosynthesis

Table S3 Significance values (P level) for correlation analysis

	pme30 33	pme00 10	MWStz 040	mws01 33	mws02 19	pme32 07
Gh_A01G072400	0.013	0.002	0.000	0.014	0.013	0.002
Gh_A02G052100	0.022	0.000	0.007	0.001	0.005	0.007
Gh_A02G091100	0.880	0.602	0.255	0.484	0.527	0.779
Gh_A02G208700	0.075	0.007	0.002	0.001	0.000	0.033
Gh_A03G134300	0.208	0.829	0.633	0.297	0.795	0.542
Gh_A03G188900	0.028	0.010	0.001	0.011	0.000	0.101
Gh_A04G117100	0.245	0.572	0.713	0.542	0.602	0.729
Gh_A05G119000	0.000	0.080	0.026	0.051	0.101	0.033
Gh_A05G154800	0.366	0.217	0.265	0.602	0.633	0.245
Gh_A05G247600	0.183	0.003	0.002	0.007	0.001	0.009
Gh_A06G220200	0.457	0.457	0.430	0.145	0.457	0.036
Gh_A07G239500	0.036	0.028	0.051	0.000	0.010	0.007
Gh_A08G040900	0.342	0.001	0.003	0.048	0.024	0.017
Gh_A08G128700	0.006	0.036	0.055	0.006	0.020	0.011
Gh_A08G172900	0.191	0.175	0.051	0.039	0.095	0.484
Gh_A12G007900	0.236	0.762	0.499	0.484	0.863	0.966
Gh_A12G024300	0.009	0.002	0.001	0.002	0.000	0.048
Gh_A12G176900	0.175	0.587	0.812	0.863	0.863	0.762
Gh_A12G266800	0.071	0.026	0.000	0.045	0.048	0.063
Gh_A13G193300	0.008	0.018	0.015	0.000	0.039	0.001
Gh_D01G104300	0.897	0.009	0.106	0.471	0.112	0.106
Gh_D01G192500	0.002	0.033	0.051	0.245	0.080	0.075
Gh_D02G166200	0.417	0.131	0.018	0.829	0.152	0.319
Gh_D02G206300	0.042	0.000	0.002	0.017	0.000	0.018
Gh_D05G048700	0.095	0.011	0.001	0.009	0.015	0.017
Gh_D05G131400	0.162	0.000	0.066	0.044	0.004	0.117
Gh_D05G181200	0.191	0.013	0.004	0.003	0.005	0.106
Gh_D05G204400	0.071	0.000	0.003	0.009	0.002	0.005
Gh_D06G170900	0.090	0.000	0.013	0.005	0.000	0.013
Gh_D08G213200	0.004	0.002	0.003	0.017	0.045	0.000
Gh_D08G239500	0.226	0.255	0.022	0.746	0.112	0.829
Gh_D09G122800	0.033	0.003	0.000	0.013	0.006	0.005
Gh_D11G214400	0.033	0.001	0.015	0.002	0.048	0.000
Gh_D11G383100	0.036	0.022	0.045	0.000	0.004	0.017
Gh_D12G023900	0.039	0.000	0.000	0.013	0.000	0.024
Gh_D13G196100	0.001	0.002	0.019	0.017	0.075	0.012
	mws10	mws00	mws14	pme30	Zmzn0	MWSmc

	50	05	78	83	00078	e119
Gh_A01G072400	0.002	0.001	0.055	0.006	0.217	0.002
Gh_A02G052100	0.000	0.005	0.026	0.002	0.010	0.001
Gh_A02G091100	0.443	0.331	0.602	0.342	0.681	0.681
Gh_A02G208700	0.001	0.001	0.112	0.003	0.159	0.000
Gh_A03G134300	0.729	0.983	0.829	0.746	0.001	0.729
Gh_A03G188900	0.001	0.000	0.124	0.003	0.090	0.000
Gh_A04G117100	0.484	0.746	0.966	0.779	0.080	0.633
Gh_A05G119000	0.036	0.101	0.665	0.159	0.106	0.011
Gh_A05G154800	0.183	0.527	0.005	0.175	0.665	0.795
Gh_A05G247600	0.010	0.000	0.014	0.003	0.319	0.007
Gh_A06G220200	0.633	0.245	0.542	0.297	0.199	0.443
Gh_A07G239500	0.015	0.009	0.199	0.031	0.003	0.007
Gh_A08G040900	0.006	0.005	0.013	0.000	0.713	0.026
Gh_A08G128700	0.022	0.015	0.199	0.124	0.001	0.010
Gh_A08G172900	0.075	0.124	0.812	0.112	0.602	0.059
Gh_A12G007900	0.931	0.484	0.513	0.443	0.033	0.846
Gh_A12G024300	0.000	0.000	0.112	0.002	0.085	0.000
Gh_A12G176900	0.602	0.966	0.863	0.443	0.095	0.829
Gh_A12G266800	0.028	0.006	0.208	0.026	0.762	0.020
Gh_A13G193300	0.002	0.042	0.118	0.055	0.002	0.008
Gh_D01G104300	0.055	0.063	0.000	0.051	0.633	0.276
Gh_D01G192500	0.067	0.045	0.499	0.055	0.286	0.018
Gh_D02G166200	0.152	0.106	0.145	0.183	0.443	0.112
Gh_D02G206300	0.000	0.000	0.009	0.001	0.124	0.000
Gh_D05G048700	0.007	0.002	0.028	0.006	0.286	0.018
Gh_D05G131400	0.004	0.002	0.004	0.008	0.075	0.030
Gh_D05G181200	0.002	0.003	0.208	0.000	0.443	0.004
Gh_D05G204400	0.000	0.002	0.014	0.000	0.124	0.000
Gh_D06G170900	0.000	0.000	0.003	0.003	0.048	0.002
Gh_D08G213200	0.010	0.003	0.138	0.015	0.118	0.004
Gh_D08G239500	0.191	0.071	0.633	0.159	0.587	0.075
Gh_D09G122800	0.001	0.002	0.022	0.002	0.379	0.002
Gh_D11G214400	0.001	0.022	0.036	0.003	0.048	0.015
Gh_D11G383100	0.007	0.008	0.183	0.008	0.013	0.007
Gh_D12G023900	0.000	0.000	0.024	0.000	0.183	0.000
Gh_D13G196100	0.003	0.032	0.117	0.073	0.034	0.015

	pme1651	pmb3101	Zmjp000182	pme0422	mws0014	pme0534
Gh_A01G072400	0.000	0.010	0.001	0.028	0.095	0.006
Gh_A02G052100	0.001	0.245	0.002	0.342	0.366	0.017
Gh_A02G091100	0.457	0.484	0.713	0.217	0.024	0.430
Gh_A02G208700	0.003	0.112	0.000	0.080	0.404	0.000
Gh_A03G134300	0.897	0.045	0.966	0.014	0.009	0.812

Gh_A03G188900	0.006	0.118	0.000	0.191	0.217	0.001
Gh_A04G117100	0.649	0.572	0.729	0.366	0.948	0.729
Gh_A05G119000	0.003	0.199	0.005	0.762	0.527	0.255
Gh_A05G154800	0.527	0.159	0.846	0.051	0.067	0.255
Gh_A05G247600	0.011	0.090	0.013	0.011	0.391	0.000
Gh_A06G220200	0.779	0.404	0.649	1.000	0.106	0.138
Gh_A07G239500	0.063	0.983	0.028	0.713	0.499	0.009
Gh_A08G040900	0.015	0.045	0.020	0.018	0.175	0.002
Gh_A08G128700	0.036	0.795	0.031	0.829	0.746	0.045
Gh_A08G172900	0.028	0.236	0.039	0.297	0.286	0.167
Gh_A12G007900	0.697	0.071	0.762	0.002	0.022	0.443
Gh_A12G024300	0.000	0.067	0.000	0.131	0.236	0.001
Gh_A12G176900	0.404	0.983	0.795	0.499	0.966	0.366
Gh_A12G266800	0.000	0.010	0.009	0.007	0.022	0.022
Gh_A13G193300	0.008	0.542	0.018	0.729	0.897	0.063
Gh_D01G104300	0.167	0.051	0.276	0.048	0.199	0.039
Gh_D01G192500	0.015	0.145	0.007	0.602	0.572	0.159
Gh_D02G166200	0.020	0.000	0.045	0.002	0.001	0.138
Gh_D02G206300	0.001	0.039	0.000	0.048	0.191	0.000
Gh_D05G048700	0.011	0.145	0.033	0.045	0.075	0.003
Gh_D05G131400	0.037	0.244	0.040	0.249	0.541	0.007
Gh_D05G181200	0.022	0.297	0.007	0.145	0.217	0.002
Gh_D05G204400	0.001	0.051	0.000	0.085	0.308	0.003
Gh_D06G170900	0.007	0.145	0.005	0.095	0.499	0.001
Gh_D08G213200	0.000	0.095	0.002	0.112	0.572	0.022
Gh_D08G239500	0.033	0.003	0.033	0.048	0.000	0.199
Gh_D09G122800	0.000	0.006	0.001	0.018	0.028	0.003
Gh_D11G214400	0.009	0.379	0.020	0.379	0.846	0.022
Gh_D11G383100	0.075	0.931	0.028	0.713	0.681	0.003
Gh_D12G023900	0.001	0.045	0.000	0.042	0.124	0.000
Gh_D13G196100	0.000	0.117	0.008	0.403	0.336	0.170

	Hmcn000192	pmb0962	mws0120	mws1090	MWSs1k045	mws0918
Gh_A01G072400	0.527	0.001	0.001	0.002	0.633	0.003
Gh_A02G052100	0.245	0.002	0.001	0.003	0.829	0.000
Gh_A02G091100	0.499	0.829	0.145	0.404	0.000	0.404
Gh_A02G208700	0.697	0.000	0.011	0.005	0.779	0.000
Gh_A03G134300	0.443	0.587	0.762	0.914	0.297	0.846
Gh_A03G188900	0.812	0.000	0.007	0.000	0.542	0.000
Gh_A04G117100	0.587	0.572	0.513	0.829	0.159	0.443
Gh_A05G119000	0.587	0.005	0.045	0.124	0.762	0.045
Gh_A05G154800	0.001	0.948	0.208	0.124	0.457	0.331
Gh_A05G247600	0.183	0.005	0.003	0.000	0.880	0.007
Gh_A06G220200	0.404	0.404	0.366	0.331	0.031	0.633

Gh_A07G239500	0.618	0.005	0.018	0.028	0.308	0.011
Gh_A08G040900	0.026	0.055	0.013	0.002	0.713	0.022
Gh_A08G128700	0.914	0.004	0.015	0.026	0.762	0.014
Gh_A08G172900	0.587	0.045	0.090	0.366	0.443	0.085
Gh_A12G007900	0.208	0.948	0.897	0.557	0.404	0.649
Gh_A12G024300	0.829	0.000	0.003	0.001	0.812	0.000
Gh_A12G176900	0.499	0.633	0.681	0.914	0.319	0.665
Gh_A12G266800	0.681	0.013	0.005	0.028	0.208	0.024
Gh_A13G193300	0.665	0.005	0.003	0.036	0.779	0.007
Gh_D01G104300	0.007	0.342	0.085	0.003	0.779	0.118
Gh_D01G192500	0.931	0.018	0.131	0.051	0.430	0.085
Gh_D02G166200	0.681	0.106	0.245	0.055	0.319	0.101
Gh_D02G206300	0.236	0.000	0.003	0.000	0.846	0.000
Gh_D05G048700	0.106	0.028	0.000	0.000	0.226	0.005
Gh_D05G131400	0.121	0.035	0.008	0.000	0.729	0.013
Gh_D05G181200	0.208	0.020	0.005	0.011	0.812	0.004
Gh_D05G204400	0.191	0.001	0.010	0.001	0.557	0.000
Gh_D06G170900	0.175	0.002	0.003	0.000	0.897	0.000
Gh_D08G213200	0.527	0.001	0.013	0.018	0.430	0.015
Gh_D08G239500	0.795	0.095	0.199	0.095	0.026	0.090
Gh_D09G122800	0.265	0.003	0.000	0.000	0.542	0.001
Gh_D11G214400	0.112	0.020	0.003	0.013	0.286	0.015
Gh_D11G383100	0.404	0.008	0.003	0.014	0.457	0.008
Gh_D12G023900	0.255	0.001	0.001	0.000	0.602	0.000
Gh_D13G196100	0.704	0.007	0.003	0.040	0.696	0.014

	Lmdp0 03110	Lmqp0 00329	MWSHY 0009	mws13 75	Lmmn0 03663	Lmsn0 00381
Gh_A01G072400	0.011	0.000	0.002	0.015	0.090	0.048
Gh_A02G052100	0.011	0.004	0.000	0.003	0.022	0.001
Gh_A02G091100	0.572	0.236	0.319	0.914	0.131	0.914
Gh_A02G208700	0.063	0.002	0.000	0.009	0.036	0.024
Gh_A03G134300	0.391	0.499	0.779	0.055	0.779	0.085
Gh_A03G188900	0.183	0.005	0.000	0.006	0.167	0.020
Gh_A04G117100	0.175	0.829	0.366	0.265	0.795	0.159
Gh_A05G119000	0.003	0.042	0.039	0.011	0.245	0.106
Gh_A05G154800	0.914	0.191	0.484	0.665	0.513	0.484
Gh_A05G247600	0.167	0.000	0.005	0.018	0.342	0.071
Gh_A06G220200	0.863	0.430	0.602	0.036	0.572	0.255
Gh_A07G239500	0.080	0.055	0.007	0.000	0.342	0.002
Gh_A08G040900	0.342	0.000	0.020	0.208	0.106	0.379
Gh_A08G128700	0.036	0.085	0.013	0.000	0.633	0.000
Gh_A08G172900	0.020	0.051	0.031	0.297	0.001	0.499
Gh_A12G007900	0.308	0.366	0.746	0.366	0.914	0.542
Gh_A12G024300	0.020	0.001	0.000	0.003	0.039	0.017

Gh_A12G176900	0.075	0.795	0.618	0.342	0.983	0.118
Gh_A12G266800	0.015	0.000	0.010	0.124	0.048	0.286
Gh_A13G193300	0.003	0.031	0.006	0.000	0.106	0.000
Gh_D01G104300	0.931	0.059	0.191	0.602	0.983	0.276
Gh_D01G192500	0.167	0.039	0.085	0.048	0.618	0.443
Gh_D02G166200	0.499	0.026	0.159	0.914	0.542	0.762
Gh_D02G206300	0.138	0.001	0.000	0.008	0.286	0.020
Gh_D05G048700	0.085	0.001	0.005	0.022	0.265	0.039
Gh_D05G131400	0.212	0.019	0.009	0.051	0.390	0.079
Gh_D05G181200	0.183	0.001	0.001	0.075	0.002	0.276
Gh_D05G204400	0.095	0.001	0.000	0.020	0.067	0.020
Gh_D06G170900	0.085	0.004	0.000	0.005	0.255	0.005
Gh_D08G213200	0.011	0.001	0.008	0.001	0.255	0.042
Gh_D08G239500	0.499	0.042	0.106	0.746	0.286	0.948
Gh_D09G122800	0.031	0.000	0.002	0.048	0.095	0.063
Gh_D11G214400	0.024	0.005	0.011	0.006	0.118	0.024
Gh_D11G383100	0.059	0.026	0.004	0.000	0.191	0.010
Gh_D12G023900	0.159	0.000	0.000	0.009	0.199	0.048
Gh_D13G196100	0.000	0.011	0.006	0.010	0.092	0.021

	Lms p004450	pmb 3107	Hmg n002833	Lmh p008440	Hmp p003270	pmb 3055	Cms p005051	Hmy p007589
Gh_A01G072400	0.005	0.000	0.001	0.005	0.404	0.366	0.015	0.762
Gh_A02G052100	0.007	0.004	0.001	0.017	0.286	0.067	0.000	0.572
Gh_A02G091100	0.746	0.649	0.572	0.812	0.587	0.297	0.484	0.199
Gh_A02G208700	0.000	0.020	0.000	0.013	0.729	0.265	0.001	0.966
Gh_A03G134300	0.914	0.746	0.779	0.457	0.366	0.226	0.713	0.059
Gh_A03G188900	0.001	0.048	0.000	0.000	0.276	0.048	0.002	0.829
Gh_A04G117100	0.983	0.812	0.762	0.829	0.004	0.001	0.513	0.846
Gh_A05G119000	0.159	0.002	0.017	0.003	0.527	0.665	0.297	0.779
Gh_A05G154800	0.379	0.513	0.697	0.746	0.729	0.966	0.145	0.618
Gh_A05G247600	0.000	0.007	0.005	0.036	0.795	0.199	0.000	0.602
Gh_A06G220200	0.286	0.199	0.527	0.276	0.342	0.880	0.226	0.001
Gh_A07G239500	0.0	0.0	0.0	0.0	0.6	0.1	0.0	0.0

	06	18	07	20	65	52	03	55
Gh_A08G040900	0.009	0.017	0.033	0.059	0.527	0.829	0.005	0.697
Gh_A08G128700	0.031	0.014	0.015	0.010	0.145	0.033	0.014	0.145
Gh_A08G172900	0.063	0.112	0.011	0.308	0.914	0.729	0.265	0.159
Gh_A12G007900	0.471	0.846	0.966	0.681	0.897	0.779	0.572	0.404
Gh_A12G024300	0.000	0.008	0.000	0.001	0.319	0.075	0.001	0.983
Gh_A12G176900	0.779	0.542	0.897	0.897	0.001	0.033	0.863	0.812
Gh_A12G266800	0.018	0.003	0.008	0.095	0.542	0.471	0.075	0.319
Gh_A13G193300	0.028	0.002	0.003	0.028	0.443	0.255	0.018	0.471
Gh_D01G104300	0.095	0.236	0.342	0.255	0.484	0.183	0.014	0.914
Gh_D01G192500	0.208	0.020	0.085	0.000	0.729	0.665	0.226	0.342
Gh_D02G166200	0.095	0.183	0.106	0.145	0.457	0.829	0.319	0.067
Gh_D02G206300	0.000	0.010	0.001	0.001	0.245	0.033	0.000	0.602
Gh_D05G048700	0.022	0.003	0.024	0.067	0.527	0.112	0.003	0.618
Gh_D05G131400	0.017	0.064	0.056	0.040	0.186	0.003	0.000	0.491
Gh_D05G181200	0.011	0.033	0.005	0.048	0.484	0.618	0.007	0.665
Gh_D05G204400	0.000	0.009	0.001	0.007	0.665	0.308	0.000	0.665
Gh_D06G170900	0.000	0.014	0.003	0.020	0.245	0.017	0.000	0.484
Gh_D08G213200	0.008	0.000	0.007	0.006	0.542	0.484	0.018	0.391
Gh_D08G239500	0.286	0.297	0.106	0.075	0.319	0.484	0.484	0.090
Gh_D09G122800	0.004	0.000	0.001	0.010	0.681	0.430	0.009	0.746
Gh_D11G214400	0.020	0.000	0.018	0.033	0.863	0.587	0.005	0.175
Gh_D11G383100	0.0	0.0	0.0	0.0	0.9	0.1	0.0	0.0

	07	11	07	28	48	67	02	51
Gh_D12G023900	0.001	0.007	0.001	0.001	0.391	0.067	0.000	0.633
Gh_D13G196100	0.070	0.000	0.016	0.032	0.106	0.103	0.043	0.991
